# Health‐Related Physical Fitness Associated With Hypertension Risk in Adults Living in Sub‐Plateau Environments

**DOI:** 10.1111/jch.70184

**Published:** 2025-11-20

**Authors:** Hao Li, Weiping Du, Cong Huang, Ming Zhang

**Affiliations:** ^1^ School of Physical Education Shaanxi Normal University Xi'an Shaanxi China; ^2^ School of Physical Education Ningxia Normal University Guyuan Ningxia China

**Keywords:** association, health‐related physical fitness, hypertension, risk of disease, sub‐plateau

## Abstract

This study aimed to investigate the associations between health‐related physical fitness (HPF) indicators and hypertension (HTN) risk among adults living in sub‐plateau regions and to explore gender‐specific differences, providing empirical evidence for cardiovascular health promotion and intervention. A cross‐sectional study was conducted from 2020 to 2022 in Ningxia, China, recruiting 3026 adults aged 20–59 years (1328 males and 1698 females). Ten HPF indicators across five dimensions, including body composition (body mass index, BMI; waist‐to‐hip ratio, WHR; waist‐to‐height ratio, WHtR), cardiorespiratory endurance (vital capacity, VC), muscular strength (grip strength, GS; back strength, BS; vertical jump, VJ), muscular endurance (push‐ups/knee push‐ups, PU/KPU; sit‐ups, SU), and flexibility fitness (sit‐and‐reach, SAR). Binary logistic regression was used to identify HTN‐related indicators, and receiver operating characteristic (ROC) analyses were performed to evaluate their predictive ability. The results showed that the prevalence of HTN was 26.75% in males, significantly higher than 18.36% in females (*p* < 0.05), both lower than the national average (males: 36.8%, females: 26.3%). Regarding the association, in males, BMI (odds ratio, OR = 1.120) and WHtR (OR = 1.673) were positively associated with HTN risk (*p* < 0.05), whereas SAR (OR = 0.975) showed a negative association (*p* < 0.05). In females, WHR (OR = 1.240) was positively associated with HTN (*p* < 0.05), while SU (OR = 0.960) showed a negative association (*p* < 0.05). ROC analysis indicated that WHtR and WHR were the best single predictors for males (area under the curve, AUC = 0.662) and females (AUC = 0.633), respectively, while combined indicators (BMI + WHtR + SAR in males; WHR + SU in females) further improved discrimination (AUC = 0.679 and 0.655). In conclusion, adults in the sub‐plateau region exhibited a lower prevalence of HTN with notable gender differences. WHtR and WHR are the most valuable gender‐specific screening indicators, and combined indices enhance predictive accuracy, offering practical guidance for early HTN prevention and management in sub‐plateau populations.

**Abbreviations**
BMI: body mass index
BS: back strength
GS: grip strength
HC: hip circumference
HPF: health‐related physical fitness
HTN: hypertension
PU/KPU: push‐ups/knee push‐ups
SAR: sit‐and‐reach
SU: sit‐ups
VC: vital capacity
VJ: vertical jump
WC: waist circumference
WHR: waist‐to‐hip ratio
WHtR: waist‐to‐height ratio

## 1 Introduction

Hypertension (HTN) is one of the most common chronic non‐communicable diseases worldwide. Its high prevalence and severe health consequences make it a significant public health concern. According to the World Health Organization (WHO), approximately 1.3 billion people globally suffer from HTN, with particularly low control rates observed in low‐ and middle‐income countries [1]. For a long time, the occurrence of HTN has been considered closely related to multiple factors, such as the environment. High‐altitude environments, due to their unique climatic conditions, variations in oxygen concentration, and residents’ long‐term adaptive physiological adjustments, exert a distinctive influence on blood pressure levels [2]. However, current studies on HTN have mainly focused on plain and plateau regions, and systematic data from sub‐plateau regions remain limited.

The sub‐plateau region, situated at an altitude of approximately 500–2200 m, features slightly lower atmospheric and oxygen partial pressures than the plains, along with a dry climate and large diurnal temperature variations. It represents a typical transitional zone in terms of climate, ecology, and physiological adaptation [3]. Although the oxygen partial pressure in this region is lower than that of the plains, it does not reach the extreme hypoxic levels of the plateau. Residents living long‐term in this mildly hypobaric and hypoxic environment may develop health characteristics that lie between the “high adaptation–low morbidity” [4] pattern of plateau populations and the “high metabolic load–high morbidity” [5] pattern of plain populations. However, in current public health research, this region remains in a dual blind spot of “insufficient high‐altitude research and inapplicable lowland models.” Most high‐altitude health studies focus on populations above 3000 m, while public health policies and screening standards are largely based on lowland populations, overlooking the unique physiological burdens and adaptive differences of sub‐plateau residents. Therefore, investigating the relationship between health‐related physical fitness (HPF) and HTN risk among sub‐plateau populations not only helps elucidate their interconnections but also provides a scientific basis for establishing precise HTN screening standards and region‐specific fitness evaluation systems—holding significant public health implications and practical value.

HPF, as an important indicator of an individual's overall health status, primarily includes five core components: body composition, cardiorespiratory endurance, muscular strength, muscular endurance and flexibility [6]. Previous studies have shown that HPF is an effective predictor of cardiovascular and metabolic disease risk [7], but in sub‐plateau environments, HPF may play an even more critical role in maintaining blood pressure homeostasis [8]. Therefore, exploring the relationship between HPF and HTN among adults in sub‐plateau regions is not only an important direction for advancing research on the etiology of HTN, but also an urgent need for developing individualized prevention strategies and reducing the burden of chronic diseases.

Based on the above, this study proposes the following hypothesis: HPF indicators of adults living in sub‐plateau regions may be significantly associated with the risk of HTN, and HPF indicators can serve as important predictors of HTN prevalence in this population. From the perspective of HPF, the study selected 10 indicators across five domains: anthropometric indicators reflecting body composition (body mass index, BMI; waist‐to‐hip ratio, WHR; waist‐to‐height ratio, WHtR); cardiorespiratory endurance indicator (vital capacity, VC); muscular strength indicators (grip strength, GS; back strength, BS; vertical jump, VJ); muscular endurance indicators (push‐up/knee push‐up, PU/KPU; sit‐up, SU); and flexibility indicator (sit‐and‐reach, SAR). The study aimed to investigate the associations between these HPF indicators and HTN among adults in sub‐plateau regions. The focus was placed on the following scientific questions: (1) To examine the impact of different HPF dimensions on HTN risk under sub‐plateau conditions. (2) To identify effective HPF indicators for assessing HTN risk among adults in this region. The ultimate goal is to provide new insights for early prevention and health management strategies for HTN in sub‐plateau populations.

## 2 Methods

### 2.1 Research Subjects

This study was conducted from 2020 to 2022 in Ningxia, China (altitude 1090–2000m) to measure and analyze HPF indicators. To ensure sample representativeness, three cities—Guyuan, Zhongwei, and Wuzhong—were selected as survey sites based on the region's geographical and altitudinal characteristics. In each city, two districts or counties were randomly selected based on urban–rural distribution and population size, resulting in a total of six survey areas. Within each survey area, 2–3 communities and administrative villages were randomly selected, and sample allocation was performed according to the proportions of urban/rural residence, gender, and age from the Seventh National Population Census of Ningxia. Finally, individual participants were selected using simple random sampling within each site. The inclusion and exclusion criteria were as follows: (1) aged 20–59 years, (2) no physical disabilities, (3) free from chronic diseases or acute conditions that could affect physical activity, and (4) exclusion of special populations such as pregnant women and professional fitness trainees. A total of 3026 participants (1328 males and 1698 females) were ultimately included in this study. The study was approved by the local Ethics Committee (Approval No. 202416057), and all participants provided written informed consent voluntarily before participating. The study protocol was conducted in accordance with the Declaration of Helsinki.

### 2.2 Measurement Methods

#### 2.2.1 HPF Indicators and Blood Pressure Measurement

HPF indicators height (m), weight (kg), waist circumference (WC, cm), and hip circumference (HC, cm) were collected to calculate anthropometric indicators of body composition reflecting HPF, including BMI, WHR, and WHtR. Height and weight were measured using an electronic sensor‐based height–weight scale. The WC and HC were measured using a non‐elastic measuring tape. For the WC, participants were required to stand upright and expose the abdominal skin, with the measurement taken at a point 1 cm above the navel. For the HC, the measurement was taken at the level of the maximum protrusion of the buttocks. Other HPF indicators were assessed according to the National Physical Fitness Measurement Standards Manual, including VC (mL), GS (kg), VJ (cm), SU (times/min), PU/KPU (times/min), SAR (cm), and BS (kg). Participants were instructed to avoid vigorous exercise within 12 h before testing and to refrain from drinking water within 30 min before the test.

Blood pressure measurement. Blood pressure was measured using an OMRON electronic sphygmomanometer (HEM‐1000) between 7:00 and 10:00 a.m. Participants were instructed to avoid consuming caffeine‐containing beverages, smoking, and engaging in vigorous physical activity for at least 30 min before the test. After sitting quietly for 5 min, blood pressure was measured on the right upper arm. Two measurements were taken, with a 1‐min interval between them, and the average value was recorded. If the difference between the two systolic blood pressure (SBP) or diastolic blood pressure (DBP) readings exceeded 5 mmHg, a third measurement was performed, and the average of all three readings was used as the final result.

It should be noted that, due to the large sample size of this study and the limitations of field personnel and testing equipment, only age was included as a covariate to control for potential confounding effects. Other relevant factors, such as family history of HTN, smoking status, alcohol consumption, dietary salt intake, physical activity level, and socioeconomic status, were not included in the model analysis due to unavailable data and are acknowledged as major limitations of this study.

#### 2.2.2 Diagnostic Criteria and Indicator Calculations

The diagnosis of HTN was based on the 2018 Chinese guidelines for the management of HTN [9]: SBP ≥ 140 mmHg and/or DBP ≥ 90 mmHg in individuals not taking antihypertensive medication. It is noted that the newly released 2024 Chinese guidelines for the management of HTN [10] include updates in diagnostic classification and risk assessment. However, relevant studies have shown that the detection rate of HTN in the 2024 edition differs little from that of the 2018 edition [11]. Therefore, considering that the data for this study were collected during 2020–2022, the use of the 2018 edition is more reasonable and ensures methodological consistency. The related anthropometric indices were calculated as follows: BMI = weight/height^2^; WHR = WC/HC; and WHtR = WC/height.

#### 2.2.3 Quality Control

Before data collection, all assessors received standardized training. All tests were conducted in strict accordance with standard protocols. Data entry was double‐entered using Excel 2016 to ensure accuracy. Before the formal statistical analyses, the sample size was estimated using PASS Version 15 (NCSS, LLC, USA). The parameters were set as follows: a two‐sided significance level of α= 0.05, statistical power (1 −β) = 0.80, and an expected effect size (odds ratio, OR) of 1.30 based on previous studies on HTN in high‐altitude populations [12, 13]. Based on previous epidemiological studies, the baseline prevalence (*P*_0_) was assumed to be 30% [14, 15]. Under these assumptions, the minimum required sample size was approximately 1003 participants per group (2006 in total). In the present study, a total of 3026 adults (1328 males and 1698 females) were included, indicating that the actual sample size met the estimation requirements and ensured adequate statistical power.

#### 2.2.4 Statistical Analysis

All statistical analyses were performed using SPSS version 26.0 (IBM, USA). First, intergroup difference testing was conducted to compare HPF indicators between hypertensive and normotensive groups, in order to preliminarily identify potentially associated variables and describe sample characteristic differences. Data normality was examined using the Kolmogorov–Smirnov test. For normally distributed variables, independent‐sample *t*‐tests were applied; for non‐normally distributed variables, Mann–Whitney *U* tests were used. To ensure consistency in data presentation, all continuous variables were expressed as median (M, Q25, Q75), while categorical variables were expressed as *n* (%), with intergroup differences analyzed using the χ^2^ test. Second, to control for potential confounding and evaluate the independent contribution of each HPF indicator, all 10 HPF variables together with age were simultaneously entered into a binary logistic regression model. Multicollinearity was assessed prior to model fitting (variance inflation factor, VIF < 10) to ensure model stability. Finally, receiver operating characteristic (ROC) curve analysis was used to evaluate the predictive performance of significant indicators, calculating the area under the curve (AUC), Youden index, optimal cutoff point, and corresponding sensitivity and specificity. ROC curves were plotted using SPSS version 26.0, with the significance level set at α= 0.05.

## 3 Results

### 3.1 General Characteristics of Hypertensive and Normotensive Participants

Table 1 shows that a total of 3026 adults participated in the HPF test, including 1328 males and 1698 females. Among them, 355 males and 312 females were identified as hypertensive. The median age was 45 years for males and 48 years for females. The prevalence of HTN was 26.75% in males and 18.36% in females, with a significantly higher rate observed in males (*p* < 0.05).

**TABLE 1:** Comparison of basic information between HTN and normal participants by gender.

Indicators	HTN		Normal	
	Male (*n* = 355)	Female (*n* = 312)	Male (*n* = 973)	Female (*n* = 1386)
Age (years)	45.00 (33.00, 52.00)^a^	48.00 (40.00, 54.00)^b^	36.00 (29.00, 48.00)	40.00 (30.00, 48.00)
Height (m)	1.71 (1.66, 1.75)	1.58 (1.54, 1.61)	1.70 (1.67, 1.74)	1.58 (1.55, 1.62)
Weight (kg)	73.20 (68.20, 78.50)^a^	60.80 (55.20, 65.80)^b^	69.60 (63.60, 74.95)	58.20 (53.40, 63.33)
BMI (kg·m^−2^)	25.25 (23.73, 26.89)^a^	24.42 (22.56, 26.17)^b^	24.07 (22.19, 25.58)	23.34 (21.33, 25.26)
WC (cm)	91.95 (86.23, 98.23)^a^	82.90 (76.40, 88.90)^b^	87.30 (81.10, 93.40)	78.65 (72.60, 84.68)
HC (cm)	99.75 (95.30, 104.03)^a^	95.10 (91.40, 99.40)^b^	97.50 (93.05, 101.50)	94.00 (90.20, 97.80)
WHR	0.93 (0.88, 0.96)^a^	0.87 (0.83, 0.91)^b^	0.89 (0.86, 0.93)	0.84 (0.80, 0.88)
WHtR	0.54 (0.50, 0.58)^a^	0.53 (0.49, 0.56)^b^	0.51 (0.48, 0.55)	0.50 (0.46, 0.54)
VC (mL)	3270.00 (2901.75, 3708.75)^a^	2178.50 (1996.25, 2400.00)^b^	3361.00 (3107.00, 3785.50)	2268.00 (2056.50, 2537.00)
GS (kg)	45.60 (41.00, 49.90)	27.30 (24.60, 30.48)	45.30 (41.50, 49.60)	27.80 (24.60, 31.20)
BS (kg)	119.75 (103.03, 134.90)	66.05 (54.43, 77.68)	117.4 (102.8, 132.9)	64.30 (52.98, 76.60)
VJ (cm)	28.90 (25.20, 35.90)^a^	21.10 (17.85, 24.95)^b^	31.60 (27.00, 36.50)	21.80 (19.00, 25.00)
SU (times/min)	21.00 (17.00, 26.00)^a^	17.00 (15.00, 20.00)^b^	22.00 (18.00, 26.00)	19.00 (16.00, 22.00)
PU/KPU (times/min)	20.00 (17.00, 24.00)^a^	20.00 (17.00, 27.00)	21.00 (17.00, 26.00)	21.00 (18.00, 27.00)
SAR (cm)	9.60 (5.60, 14.30)^a^	12.65 (8.70, 16.80)	11.10 (7.50, 15.35)	13.10 (8.78, 17.00)
SBP (mm Hg)	144.00 (137.00, 150.00)^a^	145.00 (140.00, 152.00)^b^	119.00 (110.00, 127.00)	120.00 (110.00, 127.00)
DBP (mm Hg)	93.00 (88.00, 98.00)^a^	91.00 (86.00, 98.00)^b^	71.00 (64.00, 79.00)	72.00 (65.00, 78.00)
Incidence (%)	26.75%^c^	18.36%		

^a^ indicates *p* < 0.05 vs. normal males;

^b^ indicates *p* < 0.05 vs. normal females;

^c^ indicates *p* < 0.05 for males vs. females hypertension prevalence.

After intergroup difference testing, significant differences (*p* < 0.05) were observed in the HPF indicators of BMI, WHR, WHtR, VC, VJ, SU, PU/KPU, and SAR between hypertensive and normotensive males, whereas GS and BS showed no significant differences (*p* > 0.05). Among females, significant differences (*p* < 0.05) were found in BMI, WHR, WHtR, VC, VJ, and SU, while GS, BS, PU/KPU, and SAR exhibited no significant differences (*p* > 0.05).

### 3.2 Binary Logistic Regression Analysis of HPF and HTN Risk

Before conducting logistic regression analysis, multicollinearity tests were performed on the 10 HPF indicators (BMI, WHR, WHtR, VC, GS, BS, VJ, SU, PU/KPU, and SAR) and the covariate Age in both male and female samples. The results showed that the VIF of all variables was less than 10, indicating no multicollinearity issues in the model. Next, binary logistic regression analysis was conducted, with HTN status (0 = No, 1 = Yes) as the dependent variable, age as a covariate, and the HPF indicators as independent variables (Table 2).

**TABLE 2:** Binary logistic regression results for the association between HPF indicators and HTN.

Gender	Indicators	Regression coefficient	Standard error	Wald	OR (95%CI)	*p*
Male	Age	0.034	0.008	19.208	1.035(1.019–1.051)	< 0.001
	BMI	0.102	0.033	11.864	1.120(1.050–1.194)	0.001
	WHtR	0.515	0.237	4.709	1.673(1.051–2.662)	0.030
	WHR	0.227	0.174	1.712	1.255(0.893–1.763)	0.191
	VC	0.000	0.000	0.027	1.000(1.000–1.000)	0.870
	GS	0.024	0.012	3.814	1.024(1.000–1.049)	0.051
	BS	0.000	0.003	0.010	1.000(0.994–1.006)	0.919
	VJ	0.015	0.011	1.803	1.015(0.993–1.037)	0.179
	PU/KPU	0.003	0.009	0.080	1.003(0.985–1.021)	0.777
	SU	0.015	0.012	1.461	1.015(0.991–1.039)	0.227
	SAR	−0.025	0.011	5.277	0.975(0.954–0.997)	0.022
Female	Age	0.045	0.008	32.679	1.046(1.030–1.063)	< 0.001
	BMI	0.054	0.036	2.185	1.055(0.983–1.133)	0.139
	WHtR	0.424	0.236	3.237	1.528(0.963–2.425)	0.072
	WHR	0.215	0.095	5.169	1.240(1.030–1.492)	0.023
	VC	0.000	0.000	1.299	1.000(0.999–1.000)	0.254
	GS	0.018	0.016	1.271	1.018(0.987–1.050)	0.260
	BS	0.003	0.004	0.627	1.003(0.995–1.011)	0.429
	VJ	0.016	0.015	1.250	1.016(0.988–1.046)	0.264
	PU/KPU	0.009	0.007	1.484	1.009(0.995–1.024)	0.223
	SU	−0.041	0.015	7.190	0.960(0.931–0.989)	0.007
	SAR	−0.013	0.011	1.386	0.987(0.966–1.009)	0.239

The results indicated that age was significantly associated with HTN in both males and females (*p* < 0.05). Among HPF indicators, BMI, WHtR, and SAR were significantly associated with HTN in males (*p* < 0.05) but not in females (*p* > 0.05); WHR and SU were significantly associated with HTN in females (*p* < 0.05) but not in males (*p* > 0.05). VC, GS, BS, VJ, and PU/KPU showed no significant associations with HTN in either sex (*p* > 0.05).

### 3.3 ROC Curve Analysis of HPF Indicators for Predicting HTN Risk

**FIGURE 1:**
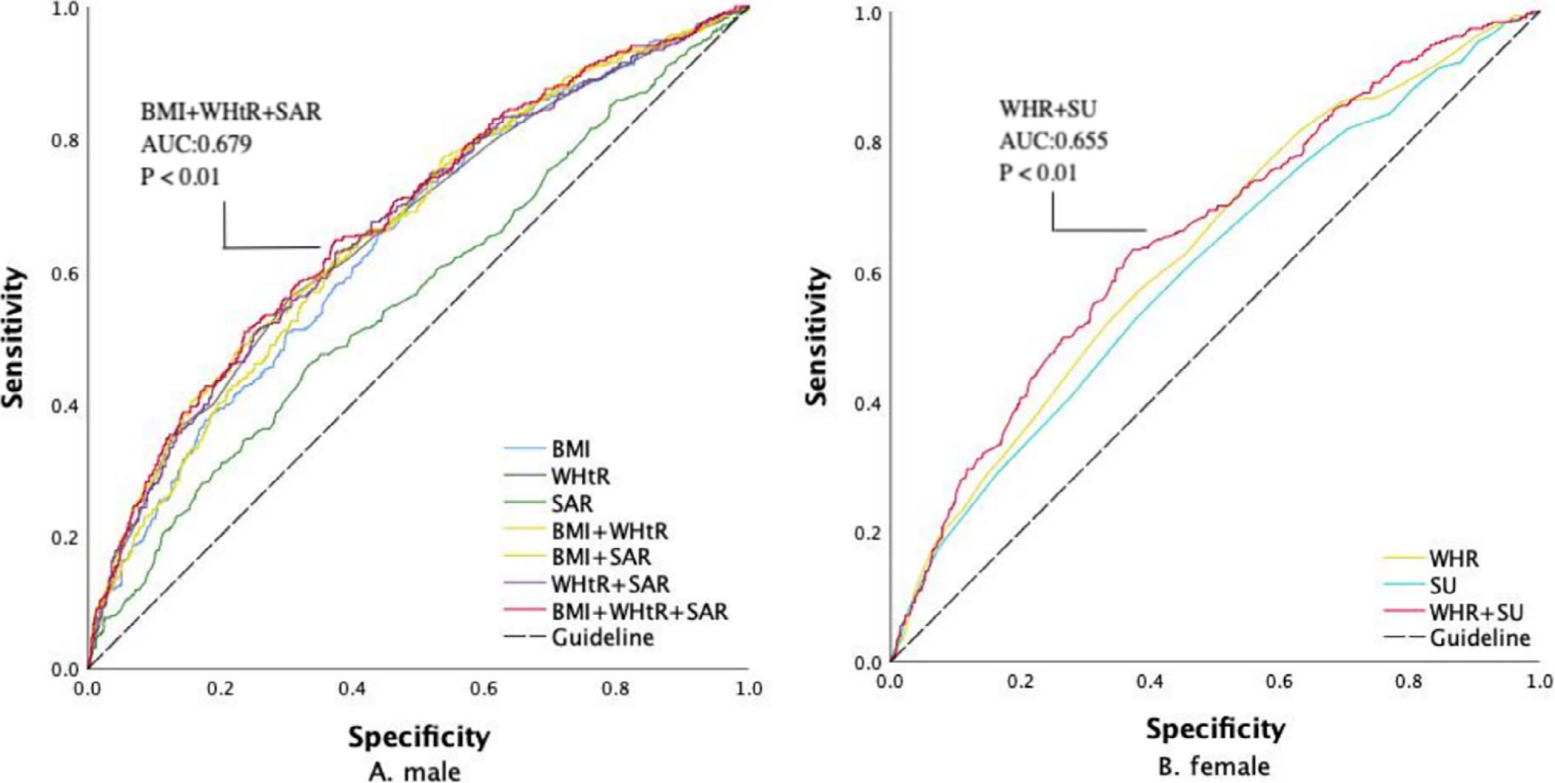
ROC curve of HPF indicators in predicting the risk of HTN.

ROC curve analysis was performed to evaluate the predictive value of individual and combined HPF indicators for HTN (Table 3, Figure 1). In males, the areas under the ROC curves (AUCs) for BMI, WHtR, SAR, BMI + WHtR, BMI + SAR, WHtR + SAR, and BMI + WHtR + SAR were 0.648, 0.662, 0.563, 0.673, 0.658, 0.667, and 0.679 (all *p* < 0.05), with BMI + WHtR + SAR showing the highest predictive value. In females, the AUCs for WHR, SU and WHR + SU were 0.633, 0.606, and 0.655 (all *p* < 0.05), with WHR + SU exhibiting the best discrimination. It should be noted that, since male SAR and female SU were significantly negatively associated with HTN prevalence, their AUC values were direction‐corrected to ensure consistent interpretability across all indicators.

**TABLE 3:** ROC curve analysis of HPF indicators for predicting HTN.

Gender	Indicators	Area under the curve (AUC)	Standard Error	95%CI	*p*
Male	BMI	0.648	0.017	0.614–0.681	< 0.001
	WHtR	0.662	0.017	0.628–0.696	< 0.001
	SAR	0.563	0.018	0.528–0.599	< 0.001
	BMI + WHtR	0.673	0.017	0.640–0.706	< 0.001
	BMI + SAR	0.658	0.017	0.625–0.691	< 0.001
	WHtR + SAR	0.667	0.017	0.634–0.701	< 0.001
	BMI + WHtR + SAR	0.679	0.017	0.646–0.712	< 0.001
Female	WHR	0.633	0.017	0.599–0.667	< 0.001
	SU	0.606	0.018	0.570–0.641	< 0.001
	WHR + SU	0.655	0.017	0.621–0.688	< 0.001

*Note*: Direction‐corrected AUC values are reported for inversely associated indicators (male SAR and female SU). The “+” symbol denotes the combined use of variables in the joint ROC curve analysis.

By calculating the Youden index for each HPF indicator and their combined indices, the optimal cutoff values for HTN were determined (Table 4). Among Males, WHtR exhibited the highest predictive performance as a single indicator (cutoff = 0.535, sensitivity = 0.562, specificity = 0.693, Youden index = 0.255). In the combined model, the optimal probability threshold for BMI + WHtR + SAR was 0.270 (sensitivity = 0.647, specificity = 0.374, Youden index = 0.273), showing the best overall predictive performance. Among Females, WHR demonstrated the highest predictive ability as a single indicator (cutoff = 0.855, sensitivity = 0.576, specificity = 0.612, Youden index = 0.188), while the combined indicator WHR + SU (cutoff = 0.188, sensitivity = 0.633, specificity = 0.373, Youden index = 0.260) exhibited superior predictive capacity. Overall, the results indicate that combined indices outperform single indicators in both genders, suggesting that multidimensional body composition assessment can more accurately the identify risk of HTN.

**TABLE 4:** Diagnostic performance of HPF indicators and combined indices for predicting HTN risk.

Gender	Indicators	Cutoff point	Sensitivity	Specificity	Youden's Index
Male	BMI	23.96	0.734	0.486	0.220
	WHtR	0.535	0.562	0.693	0.255
	SAR	8.85	0.677	0.544	0.123
	BMI + WHtR	0.314	0.508	0.251	0.257
	BMI + SAR	0.258	0.662	0.424	0.238
	WHtR + SAR	0.309	0.506	0.251	0.255
	BMI + WHtR + SAR	0.270	0.647	0.374	0.273
Female	WHR	0.855	0.576	0.612	0.188
	SU	18.50	0.541	0.391	0.150
	WHR + SU	0.188	0.633	0.373	0.260

*Note*: The cutoff value was determined based on the maximum Youden index. For combined indices, the “cutoff point” represents the optimal probability threshold derived from logistic regression models based on the maximum Youden index.

## 4 Discussion

### 4.1 Prevalence of HTN Among Adults in Sub‐Plateau Areas

In this study, the prevalence of HTN was 26.8% among males and 18.4% among females, both lower than the national averages reported for Chinese adults during 2020–2022 (36.8% for males and 26.3% for females) [16]. Similar findings have been reported in high‐altitude regions such as Peru, Nepal, and parts of Latin America, where residents show lower HTN prevalence than those at sea level [17, 18, 19]. This phenomenon may be attributed to a comprehensive set of cardiovascular protective adaptations developed under chronic mild hypoxia. First, long‐term hypoxia promotes continuous endothelial release of vasodilators such as nitric oxide (NO) and prostaglandins, reduces responsiveness to vasoconstrictors like angiotensin II and endothelin‐1, and suppresses renin–angiotensin–aldosterone system activity, thereby lowering peripheral vascular resistance and maintaining reduced arterial pressure [20]. Second, sub‐plateau residents exhibit lower resting sympathetic activity and higher vagal tone, forming more stable regulation of heart rate and blood pressure compared with the transient sympathetic activation seen in lowlanders newly exposed to altitude [21]. Third, circulatory adaptations—such as slight plasma volume reduction and increased capillary density—enhance oxygen delivery without elevating blood pressure [22]. Additionally, mild hypoxia suppresses appetite and increases basal metabolic rate; combined with higher physical activity and lower obesity prevalence, this leads to healthier body composition and further reduces HTN risk [23]. Together, these physiological adaptations may underlie the relatively low HTN prevalence observed among sub‐plateau populations.

In addition, the gender difference in HTN prevalence may result from multiple factors. Biologically, males and females differ inherently in cardiovascular regulation. Studies have shown that males tend to exhibit higher sympathetic nerve activity, elevated renin–angiotensin–aldosterone system (RAAS) activity, and greater vascular reactivity, all closely linked to HTN development [24, 25]. In sub‐plateau environments, these differences may be amplified by mild hypoxia. Long‐term sub‐plateau exposure has been found to maintain higher HTN risk in males due to sustained sympathetic and RAAS hyperactivity and altered vascular calcium regulation [26]. From a behavioral perspective, males have higher exposure rates to unhealthy lifestyle factors—such as smoking, alcohol consumption, and high‐salt or high‐fat diets—and are more prone to stress‐related responses, further increasing their HTN risk [27].

### 4.2 Association Between HPF Indicators and HTN Risk

This study found that BMI and WHtR were positively associated with HTN risk among males, whereas SAR showed a negative association, suggesting that HTN risk in sub‐plateau males is mainly influenced by abdominal fat accumulation and arterial compliance. The positive relationship between BMI and HTN aligns with previous high‐altitude research. For example, Song et al. reported that although residents of the Tibetan Plateau have lower average BMI than those living at lower altitudes, BMI remains significantly positively correlated with blood pressure and HTN prevalence [4]. Similarly, Palmer et al. noted that while high‐altitude environments—characterized by hypoxia, reduced appetite, increased energy expenditure, and dietary differences—tend to lower overall BMI levels, BMI remains an important individual‐level indicator of HTN risk [23]. In terms of predictive performance, WHtR outperformed BMI in identifying HTN risk among males. Studies conducted in Tibetan regions have shown that abdominal obesity is more strongly associated with HTN than overall obesity [28], suggesting that height‐standardized central obesity indicators (such as WHtR) may serve as more sensitive predictors under altitude exposure. This result is further supported by large‐scale studies in the general Chinese male population, which also found WHtR to be superior to BMI in predicting HTN risk [29], consistent with the present findings. Moreover, SAR was significantly and inversely associated with HTN risk, indicating that lower flexibility may reflect greater arterial stiffness. Studies have shown that four weeks of static stretching training can effectively reduce arterial stiffness, and flexibility is inversely associated with central arterial pressure and pulse wave velocity [30]. Systematic reviews have further confirmed that individuals with better flexibility generally exhibit higher arterial compliance [31]. These findings provide vascular‐level support for the present study's conclusion that “lower SAR is associated with higher HTN risk.”

In contrast, no significant associations were found between other HPF indicators in males (WHR, VC, GS, BS, VJ, SU, and PU/KPU) and HTN risk. Although WHR also reflects abdominal obesity, its denominator, HC, is considered metabolically protective [32]. Because males tend to accumulate fat in the abdomen, HC varies less and offers limited protection, making WHR less stable than WHtR in indicating abdominal fat–related risk. This may explain why WHR was nonsignificant while WHtR showed stronger discriminative power. As a pulmonary function indicator, VC is generally higher among high‐altitude residents [33], reflecting long‐term physiological adaptation to hypoxia. This elevation likely reduced variability within the sample (“ceiling effect”), weakening its cross‐sectional association with blood pressure. Moreover, the intrinsic correlation between lung function and blood pressure is modest, which may account for the nonsignificant findings among sub‐plateau males with uniformly high VC levels [34]. The relationship between GS and HTN has shown heterogeneity across studies. Some have reported no significant association between absolute GS and HTN [35], whereas relative GS (adjusted for body weight or BMI) shows a more consistent negative association [36]. In this study, absolute GS yielded a near‐significant result (*p* = 0.051), supporting its instability as a predictive marker. Few studies have examined BS and HTN; Cavazz et al. [37] similarly found no significant association in a working population, consistent with our findings. Evidence for VJ and HTN is also limited in cross‐sectional research. Although improving VJ performance may reduce blood pressure, this “training–function–BP” pathway is easily confounded by factors such as body fat distribution, masking its independent effect [38]. SU did not effectively predict HTN risk, likely due to test characteristics. SU performance mainly reflects local muscular endurance but is affected by compensatory movement, abdominal fat thickness, and trunk proportions [39]. In males with greater abdominal fat, higher body weight, or fat mass may reduce repetitions, confounding the effects of muscular endurance and body composition on BP. Similarly, PU/KPU showed no significant association with HTN. A US male cohort study reported that higher bench‐press strength was linked to reduced HTN risk in the “pre‐hypertensive” subgroup, but this association disappeared after adjusting for aerobic fitness, suggesting that muscular strength effects may be overshadowed by stronger correlates [40]. Moreover, as a bodyweight‐dependent test, obese individuals tend to complete fewer repetitions due to excess load, while obesity itself increases HTN risk—potentially creating a spurious “fewer repetitions–higher BP” relationship.

Among females, WHR was positively associated with HTN risk, while SU was negatively associated, suggesting that fat distribution and core muscular endurance may play important roles in female HTN risk. The positive WHR–HTN relationship aligns with existing evidence that central fat distribution is a more sensitive indicator of metabolic and vascular risk [41]. This association may be influenced by gender differences in fat distribution and menopausal status. Generally, males tend to accumulate abdominal fat, whereas females—especially premenopausal women—store more subcutaneous fat in the gluteofemoral region. After menopause, declining estrogen levels lead to a shift toward abdominal fat accumulation [42]. These differences mean that the same circumference indices carry different implications across sexes. In females, HC itself exerts a protective metabolic effect—greater HC is linked to lower cardiometabolic risk, as gluteofemoral fat can sequester free fatty acids and release a more favorable adipokine profile. Thus, an elevated WHR (reflecting increased waist or reduced HC) simultaneously indicates higher abdominal fat burden and loss of gluteal protection, making its association with blood pressure more sensitive and pronounced. This may explain the positive WHR–HTN relationship observed in this study. The negative association between SU and HTN showed some inconsistency with previous findings. A large urban community study of women (*n* = 9216) found that after adjusting for age, alcohol intake, and exercise intensity, the SU–HTN relationship was not statistically significant [43]. This suggests that in females, the cross‐sectional link between muscular endurance and blood pressure may vary across populations and be easily affected by confounding factors. Moreover, the validity of SU as an abdominal endurance measure may differ among populations, which could dilute or obscure its true association in female samples and partially explain the heterogeneity across studies.

In contrast, no significant associations were observed between BMI, WHtR, VC, GS, BS, VJ, PU/KPU, or SAR and HTN risk among females. Although BMI reflects overall adiposity, it does not capture fat distribution. HTN is more closely linked to abdominal fat, whereas females typically store more subcutaneous fat in the gluteofemoral region, which is metabolically protective [32]. Thus, at the same BMI, females generally have less visceral fat than males, which may explain the lack of association between BMI and HTN. The nonsignificant relationship between WHtR and HTN in sub‐plateau females may be related to female‐specific fat distribution and age structure. The mean age of female HTN cases in this study was 48 years, corresponding to the early perimenopausal stage, when estrogen fluctuations drive fat redistribution from gluteofemoral to abdominal areas, resulting in high individual variability. Consequently, WHtR may not accurately reflect visceral fat among females at this stage, weakening its predictive power. Previous studies have confirmed that WHtR better predicts HTN risk in postmenopausal females [42], indirectly supporting this finding. Similarly, the absence of significant associations between VC, GS, BS, and VJ and HTN in females may share explanations with those observed in males. Regarding SAR, the lack of association can be interpreted from both statistical and physiological perspectives. Statistically, females generally exhibit greater flexibility, resulting in a narrower SAR distribution and reduced variability, which limits the power to detect real associations. Physiologically, most female participants (mean age 48 years) had not yet fully transitioned to postmenopause, and estrogen's protective effects on vascular elasticity remained partially intact [44], which may have attenuated or masked the independent contribution of flexibility to HTN risk.

### 4.3 Predictive Performance of HPF Indicators for HTN Risk

ROC analysis in this study showed that among males, BMI, WHtR, and their combined indicators demonstrated moderate predictive power, with WHtR performing best. This suggests that abdominal obesity better reflects HTN risk than general obesity, consistent with prior findings that WHtR provides more stable and cross‐population prediction than BMI [4, 29]. Although BMI may be influenced by body weight reduction at high altitudes, it still showed strong predictive ability in sub‐plateau males, indicating that overall body mass remains an important metabolic basis for HTN development [23]. SAR showed a weaker but inverse relationship, implying a potential link between flexibility and arterial stiffness. Previous studies have shown that flexibility training can improve vascular elasticity and reduce central arterial pressure, thus delaying blood pressure elevation [30, 31]. While SAR alone had limited predictive value, combining it with body composition indicators added information on vascular health. Notably, the combined model of BMI + WHtR + SAR achieved the highest discriminative ability, outperforming any single indicator. The combinations of BMI + WHtR and WHtR + SAR also performed well, suggesting that multi‐indicator models capture both metabolic (abdominal obesity) and functional (vascular elasticity) pathways of HTN risk [45, 46]. Among the individual HPF indicators in females, WHR showed the best performance, indicating that fat distribution remains an important risk factor for HTN in females. SU was weakly and inversely associated with HTN, suggesting that core muscular endurance may exert a protective effect by maintaining vascular and autonomic function. The combined WHR + SU model performed best, outperforming any single indicator, suggesting that integrating fat distribution and muscular indicators provides a more comprehensive assessment of HTN risk in females. This finding aligns with previous studies emphasizing that female cardiovascular risk is jointly influenced by fat distribution and muscle mass [47], and further highlights the importance of multi‐indicator assessment in female populations.

The optimal cutoff values determined by the maximum Youden index were BMI (23.96 kg/m^2^) and WHtR (0.535) for males. The BMI threshold was close to the Chinese metabolic warning standard (≈24 kg/m^2^) [48], and the WHtR cutoff exceeded the commonly used 0.5 threshold [49], indicating good practical applicability. The BMI + WHtR + SAR combination had the highest Youden index, showing improved identification of high‐risk individuals. For females, the main cutoffs were WHR = 0.855 and SU = 18.5 times/min, with the WHR close to the Chinese female health risk threshold (≈0.85) [50], suggesting its utility for rapid HTN risk screening. The WHR + SU model performed best, reflecting the complementary effects of fat distribution and core muscular endurance. Overall, ROC and cutoff analyses indicate that under sub‐plateau conditions, male HTN prediction primarily depends on body fat parameters and trunk flexibility, while female risk identification relies more on fat distribution and core muscular endurance. The combined use of multiple HPF indicators holds promise for developing a multidimensional health risk screening system tailored to sub‐plateau populations.

## 5 Conclusion

This study revealed that the prevalence of HTN among adults in the sub‐plateau region (Ningxia) was lower than the national average and showed significant sex differences. In males, BMI, WHtR, and SAR, and in females, WHR and SU, were identified as the main predictive indicators of HTN risk. WHtR and WHR served as the optimal gender‐specific screening factors, while combined multi‐indicator models further improved risk identification efficiency. This study provides both theoretical and empirical support for establishing an HPF–based early screening and risk assessment system for HTN in sub‐plateau populations and offers scientific guidance for developing targeted exercise intervention strategies.

## 6 Limitations and Future Directions

Although this study preliminarily explored the relationship between HTN risk and HPF among adults in sub‐plateau regions, it was limited by available survey resources and implementation conditions. Several potential HTN risk factors—such as family history, smoking status, alcohol consumption, dietary salt intake, physical activity level, and socioeconomic status—were not controlled for. In addition, the absence of longitudinal data prevented verification of the dynamic evolution of threshold values.

Future studies should establish a multi‐center, longitudinal health database for sub‐plateau populations and adopt an altitude‐gradient design to explore dynamic thresholds of indicators such as WHtR and WHR across altitude levels and between sexes. Moreover, machine learning algorithms should be used to construct multidimensional HTN risk prediction models integrating HPF indicators, environmental exposure, and genetic factors. The development of portable screening tools based on HPF indicators could further provide scientific support for regional public health policymaking and individualized health management.

## Author Contributions

**Hao Li**: writing – original draft, writing – review and editing, conceptualization, investigation, data curation, formal analysis, methodology, project administration, software, validation, visualization, resources. **Weiping Du**: writing – original draft, writing – review and editing, conceptualization, methodology, project administration, visualization. **Cong Huang**: writing – original draft, writing – review and editing, conceptualization, formal analysis, project administration, funding acquisition, supervision. **Ming Zhang**: writing – original draft, writing – review and editing, conceptualization, methodology, funding acquisition, project administration, visualization.

This study was supported by the Natural Science Foundation of Ningxia (Grant No. 2025AAC030630).

## Ethics Statement

The study was approved by the local ethics committee (Approval No. 202416057). All participants voluntarily took part in the study and provided written informed consent. All procedures were conducted in accordance with the principles of the Declaration of Helsinki and national ethical regulations. No direct contact with participants occurred, and all analyses were performed in a manner that ensured individual privacy and confidentiality.

## Conflicts of Interest

The authors declare no conflicts of interest.

## Data Availability

The raw data described in this study is submitted as an attachment.
